# Wristwatch PCR: A Versatile and Efficient Genome Walking Strategy

**DOI:** 10.3389/fbioe.2022.792848

**Published:** 2022-04-12

**Authors:** Lingqin Wang, Mengya Jia, Zhaoqin Li, Xiaohua Liu, Tianyi Sun, Jinfeng Pei, Cheng Wei, Zhiyu Lin, Haixing Li

**Affiliations:** ^1^ State Key Laboratory of Food Science and Technology, Nanchang University, Nanchang, China; ^2^ Sino-German Joint Research Institute, Nanchang University, Nanchang, China; ^3^ Charles W. Davidson College of Engineering, San Jose State University, San Jose, CA, United States; ^4^ Key Laboratory of Poyang Lake Environment and Resource Utilization, Ministry of Education, School of Environmental and Chemical Engineering, Nanchang University, Nanchang, China

**Keywords:** wristwatch primer, partially annealing, wristwatch-like DNA, wristwatch PCR, genome walking

## Abstract

Genome walking is a method used to retrieve unknown flanking DNA. Here, we reported wristwatch (WW) PCR, an efficient genome walking technique mediated by WW primers (WWPs). WWPs feature 5′- and 3′-overlap and a heterologous interval. Therefore, a wristwatch-like structure can be formed between WWPs under relatively low temperatures. Each WW-PCR set is composed of three nested (primary, secondary, and tertiary) PCRs individually performed by three WWPs. The WWP is arbitrarily annealed somewhere on the genome in the one low-stringency cycle of the primary PCR, or directionally to the previous WWP site in one reduced-stringency cycle of the secondary/tertiary PCR, producing a pool of single-stranded DNAs (ssDNAs). A target ssDNA incorporates a gene-specific primer (GSP) complementary at the 3′-end and the WWP at the 5′-end and thus can be exponentially amplified in the next high-stringency cycles. Nevertheless, a non-target ssDNA cannot be amplified as it lacks a perfect binding site for any primers. The practicability of the WW-PCR was validated by successfully accessing unknown regions flanking *Lactobacillus brevis* CD0817 glutamate decarboxylase gene and the hygromycin gene of rice. The WW-PCR is an attractive alternative to the existing genome walking techniques.

## Introduction

Genome walking refers to a cluster of molecular technologies that are used to capture the full-length sequence of a target gene or identify unknown regions adjacent to a known sequence. Genome walking is particularly useful when the genetic information available for biological sequence analysis is limited (Leoni et al., 2011; Ashrafmansouri et al., 2020). Genome walking relies on genome library screening or the PCR. However, the construction and screening of genomic DNA libraries are cumbersome and labor-intensive (Li et al., 2015; Zeng et al., 2020). Hence, PCR-based methods are currently promising as they are considered simple and rapid. Although numerous, the reported PCR-based walking techniques can be classified into three types according to the involved rationales (Wang et al., 2007; Chang et al., 2018; Alquezar-Planas et al., 2020): 1) inverse PCR (Ochman et al., 1988), 2) cleavage-ligation-mediated PCR (Mueller et al., 1989; Rosenthal et al., 1990; Jones et al., 1992), and 3) randomly primed PCR (Liu et al., 1995; Tan et al., 2005; Wang et al., 2013).

In the inverse PCR, a circularized target DNA must be generated by digesting genomic DNA, followed by intramolecular ligation. Then, the fragments of interest upstream and downstream from a known sequence are amplified by two GSPs with inverse extension directions (Ochman et al., 1988; Huang 1994; Ji et al., 2010). Although the specificity of the inverse PCR is high, its efficiency is restricted due to the limited quantity and size of circularized target DNA (Tan et al., 2005; Uchiyama et al., 2006). In the cleavage-ligation-mediated PCR, digested genomic DNA is ligated with an adapter/linker/cassette DNA. A target segment is then enriched by successive PCRs conducted by the adapter/linker/cassette primer sequentially pairing with nested GSPs (Dawes et al., 2020; Tran et al., 2021). A large target fragment may be obtained by this method, but it suffers from background resulting from the adapter/linker/cassette primer (Yan et al., 2003; Tan et al., 2005; Ji et al., 2010). Restriction cleavage and subsequent DNA ligation are compulsory in these two PCR methods. Additionally, the genomic DNA quality profoundly affects experimental outcomes (Terauchi et al., 2000; Li et al., 2019).

The randomly primed PCR is relatively of low-cost and straightforward as it avoids restriction and ligation steps (Jia et al., 2017; Hu et al., 2019). The thermal asymmetric interlaced PCR (TAIL-PCR), partially overlapping primer-based PCR (POP-PCR), and self-formed adaptor PCR (SFA-PCR) represent randomly primed approaches (Sessions et al., 2002; Settles et al., 2004; Zhang et al., 2018). Nevertheless, the length of the sequence obtained by the TAIL-PCR is often less than satisfactory (Yan et al., 2003; Tan et al., 2005; Li et al., 2015). In addition, in the TAIL-PCR, high background arising from the short walking primer is inevitable (Thirulogachandar et al., 2011; Yu et al., 2020). For the SFA-PCR, the walking primer is not universal, and a high concentration of DNA template is required to facilitate the generation of the panhandle-like structure (Wang et al., 2007). The POP-PCR is efficient but requires many walking primers which complicate experimental operations (Li et al., 2015; Chang et al., 2018; Yik et al., 2021).

Herein, we proposed the wristwatch (WW) PCR, a method based on the wristwatch-like structure formed between walking primers, to obtain unknown flanks. We devised three walking primers having a 5′- and 3′-overlap and a middle mismatch. Clearly, any two walking primers can form a wristwatch-like structure under sufficiently low temperature. The walking primer is thus called the wristwatch primer (WWP). A WWP set selectively enriches target DNA and simultaneously excludes the undesired DNA due to the fact that in each PCR step, the one low-/reduced-stringency cycle restricts partial annealing of any primer to only one. The feasibility of this method was verified by isolating flanks of the glutamate decarboxylase (*gadA*) locus and hygromycin gene (*hyg*). The WW-PCR can be used to probe unknown DNA flanks, identify transgene integration sites, and obtain new genes from environmental DNA.

## Materials and Methods

### Genomic DNA Isolation

The genomic DNA of *Lactobacillus brevis* CD0817 was extracted with the Bacterial Genomic DNA Isolation Kit (TIANGEN Biotech Co., Ltd., Beijing, China), according to the manufacturer’s guidance. Rice genomic DNA was kindly supplied by Dr. Xiaojue Peng (Nanchang University).

### Primers

The oligonucleotide sequences of all WWPs are completely random and are 25 nucleotides (nt) in length comprising the identical 5′- (12 nt) and 3′-part (3 nt) and the mutually mismatched spacer (10 nt). Any WWP has a high melting temperature (60–65°C) and an even distribution of the four bases (A, T, C, and G). The annealing temperature between the WWPs is approximately 40°C. An obvious self-dimer or hairpin should be avoided for any WWP. Other rules in designing WWPs are consistent with those for a regular primer. Nested GSPs were selected according to the *gadA* locus (GenBank accession number AYM03982.1) of *L. brevis* CD0817 or the *hyg* gene (KF206149.1) of rice. A GSP has a similar melting temperature with its paired WWP. All primer pairs are free of the obvious primer dimer (Table 1).

**TABLE 1:** Primers used in this study.

Primer	Oligo sequence
WWP1	CGT​CTC​CAG​TCTCCA​TGT​GTT​CGTC
WWP2	CGT​CTC​CAG​TCTTAG​GCA​CAG​TGTC
WWP3	CGT​CTC​CAG​TCTAGT​CAG​TCA​GGTC
*gadA*GSP1	TCC​ATA​CCC​TCA​TCT​CCA​TTT​CCA​T
*gadA*GSP2	AAC​TAT​CAC​CCC​ACA​ACG​TCA​TCT​C
*gadA*GSP3	ACC​GTT​CAT​AGG​CGA​AAT​TGT​TTG​T
*hyg*GSP1	CGG​CAA​TTT​CGA​TGA​TGC​AGC​TTG​G
*hyg*GSP2	CGG​GAC​TGT​CGG​GCG​TAC​ACA​AAT​C
*hyg*GSP3	GAC​CGA​TGG​CTG​TGT​AGA​AGT​ACT​C

Note: The WWPs possess identical 5′- (12 nt) and 3′-end parts (3 nt)and a heterologous spacer (10 nt) (underlined). WWP: wristwatch primer.

### PCR Procedures

**FIGURE 1:**
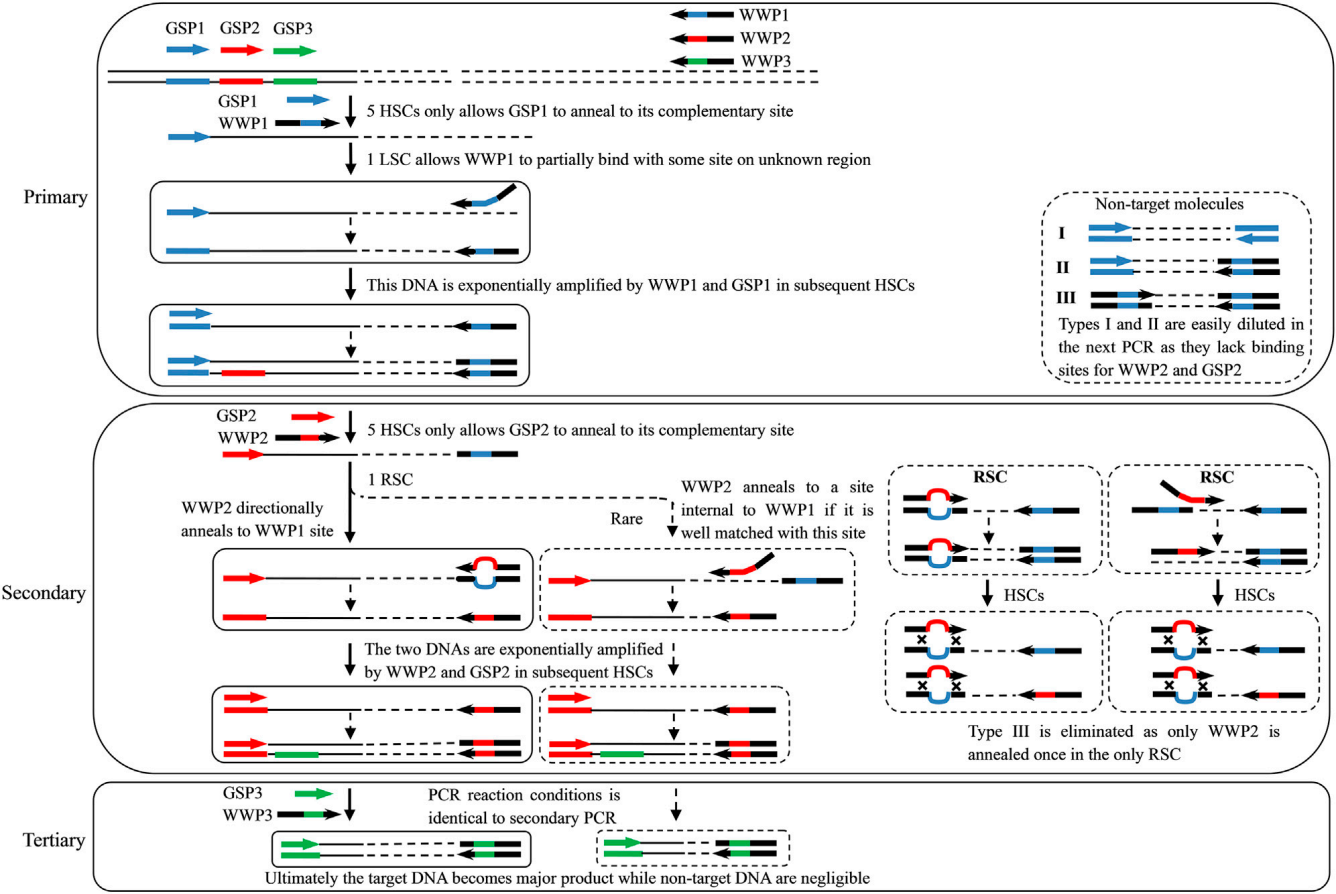
Overview of the wristwatch PCR. GSP1, GSP2, and GSP3: gene-specific primers for primary, secondary, and tertiary PCRs, respectively; WWP1, WWP2, and WWP3: wristwatch primers for primary, secondary, and tertiary PCRs, respectively; thin solid line: known sequence; thin dotted line: unknown sequence; colorful thick line: primer complement; HSC, high-stringency cycle; LSC, low-stringency cycle; and RSC, reduced-stringency cycle. Only the WWP permutation WWP1-WWP2-WWP3 is presented here to illustrate the wristwatch PCR. The sequence of any WWP is random. However, WWPs can anneal to each other’s complements in the RSC attributed to the partial overlap (as shown in Table 1), thus generating a wristwatch-like structure.

Three permutations were produced from the three WWPs, which were respectively paired with three nested GSPs to conduct three sets of WW-PCRs, as shown in Table 2. Three successive rounds (primary, secondary, and tertiary) of PCRs were proceeded in each WW-PCR set (Figure 1). In the primary PCR, genomic DNA was used as a template, and in the secondary or tertiary PCR, the previous PCR product was used as a template. The primary PCR reaction mixture in a volume of 50 μL contained 5 μL of 10 × LA PCR buffer II (Mg^2+^ plus), 8 μL of dNTP mixture (2.5 mM each), 1 μL of each primer (10 μM each), 1 μL of genomic DNA (10–100 ng for *L. brevis* CD0817 and 100–1000 ng for rice), and 0.5 μL of TaKaRa LA Taq polymerase (5 U/μL). The secondary/tertiary PCR reaction mixture (50 μL) incorporated 5 μL 10 × LA PCR buffer II (Mg^2+^ plus), 8 μL of dNTP mixture (2.5 mM each), 1 μL of each primer (10 μM each), 1 μL of the former PCR product, and 0.5 μL of TaKaRa LA Taq polymerase (5 U/μL). Each round of PCR consisted of three annealing stages: stage 1, five high-stringency (65°C) cycles (HSC); stage 2, one low-stringency (25°C) cycle (LSC) in the primary PCR or one reduced-stringency (40°C) cycle (RSC) in secondary/tertiary PCR; and stage 3, 25 HSCs (65°C). The detailed thermal cycling parameters for the WW-PCR are presented in Table 3.

**TABLE 2:** Pairing of the WWP permutation with the GSP set in nested PCRs.

Round of PCR	WWP permutation			GSP primer set
Primary	WWP1	WWP2	WWP3	GSP1
Secondary	WWP2	WWP3	WWP1	GSP2
Tertiary	WWP3	WWP1	WWP2	GSP3

Note: The three WWPs are respectively paired with the GSP, in the same row to perform three parallel WW-PCRs. A WWP permutation is shown in the same column.

**TABLE 3:** Thermal cycling parameters of the WW-PCR.

Round of PCR	Stage	Thermal condition	Cycle number
Primary		94°C, 3 min	
	1	94°C 30 s, 65°C 30 s, and 72°C 2 min	5
	2	94°C 30 s, 25°C 30 s, and 72°C 2 min	1
	3	94°C 30 s, 65°C 30 s, and 72°C 2 min	25
		72°C 3 min	
	1 μL of the primary product is directly used as the template for the secondary PCR		
Secondary		94°C, 3 min	
	1	94°C 30 s, 65°C 30 s, and 72°C 2 min	5
	2	94°C 30 s, 40°C 30 s, and 72°C 2 min	1
	3	94°C 30 s, 65°C 30 s, and 72°C 2 min	25
		72°C 3 min	
	1 μL of the secondary product is directly used as the template for the tertiary PCR		
Tertiary	Thermal cycling profile of the tertiary PCR is identical to that of the secondary PCR		

### DNA Manipulation and Sequencing

PCR products were purified using the TaKaRa MiniBEST Agarose Gel DNA Extraction Kit Ver.4.0 (Dalian, China). A purified fragment was ligated to pMD19-T simple vector using the T-vector Kit (TaKaRa). Then, the recombinant plasmids were transformed into *E. coli* DH5α cells in accordance with the instruction of TaKaRa. Several selected positive colonies were then sequenced by Shanghai Sangon Biotech Co., Ltd. (Shanghai, China).

## Results

### Overview of Wristwatch-PCR

Each walking includes three parallel sets of WW-PCRs individually performed by the three WWP permutations WWP1-WWP2-WWP3, WWP2-WWP3-WWP1, and WWP3-WWP1-WWP2 (Table 2). Each WW-PCR set consists of three successive rounds (primary, secondary, and tertiary) of nested PCRs. For convenience and clarity, only permutation WWP1-WWP2-WWP3 is employed to illustrate the rationale and process of the WW-PCR (Figure 1).

In the primary PCR, the first five HSCs only allow GSP1 to anneal to its complementary site on a known region, thus authentically increasing copies of target single-stranded DNA (ssDNA). The following one LSC makes WWP1 arbitrarily anneal to a certain place(s) on an unknown region of ssDNA and extends toward GSP1 and other loci. As a result, an ssDNA pool comprising target and non-target molecules is newly generated. It should be emphasized that the 10 nt internal mismatch allows the WWPs anneal to distinctive loci on the unknown flanking region. If more than one WWP is used in parallel, at least one will successfully anneal to the flanking region. In the next one HSC, a nascent target ssDNA is converted into double-stranded DNA (dsDNA) defined by GSP1 and WWP1 as it has an exact binding site for GSP1 at the 3′-end; this dsDNA can be exponentially enriched in the remaining HSCs. A non-target ssDNA, however, cannot be converted into dsDNA in the HSCs because it lacks perfect binding sites for any primers and thereafter is diluted.

In the secondary PCR, the first five HSCs only permit GSP2 to hybridize to its complement on known regions and extend toward WWP1, thus accumulating the ssDNA of interest. In the next one RSC, WWP2 directionally anneals to the WWP1 locus to form a wristwatch-like structure (if WWP2 matches some site(s) internal to the WWP1 well, WWP2 annealing to this site cannot be ruled out) and then initiates DNA elongation (Figure 1). As a result, a pool of ssDNAs is newly produced. The nascent target ssDNA has WWP2 at the 5′-end and the GSP2 complement at the 3′-end, which is converted into dsDNA driven by GSP2 in the next one HSC. This dsDNA is exponentially amplified in the remaining HSCs. The non-target ssDNA, however, cannot be converted into the double-stranded form in the HSCs due to the absence of a perfect binding site for any primers. This non-target ssDNA is hence removed.

The tertiary PCR driven by GSP3 and WWP3 is used to further eliminate non-target products; the involved mechanism and process are the same as those of the secondary PCR. Eventually, the target molecule becomes predominant.

### Genome Walking of *gadA* and *hyg*

To verify the feasibility of the WW-PCR, we applied this method to isolate lateral segments of *L. brevis* CD0817 *gadA* and rice *hyg*. The *L. brevis* CD0817 genome (AYM03982.1), as well as rice *hyg* (KF206149.1), and its surrounding region were deposited in the GenBank database. A portion of *gadA* or *hyg* was designated as a known sequence for designing nested GSPs, and the region adjacent to the known DNA was assumed to be an “unknown sequence”, which are herein collectively referred to as the reference sequence. The three WWP permutations (WWP1-WWP2-WWP3, WWP2-WWP3-WWP1, and WWP3-WWP1-WWP2) were used to perform three parallel sets of WW-PCRs in each walking, by pairing with a GSP set (Table 2), respectively.

**FIGURE 2:**
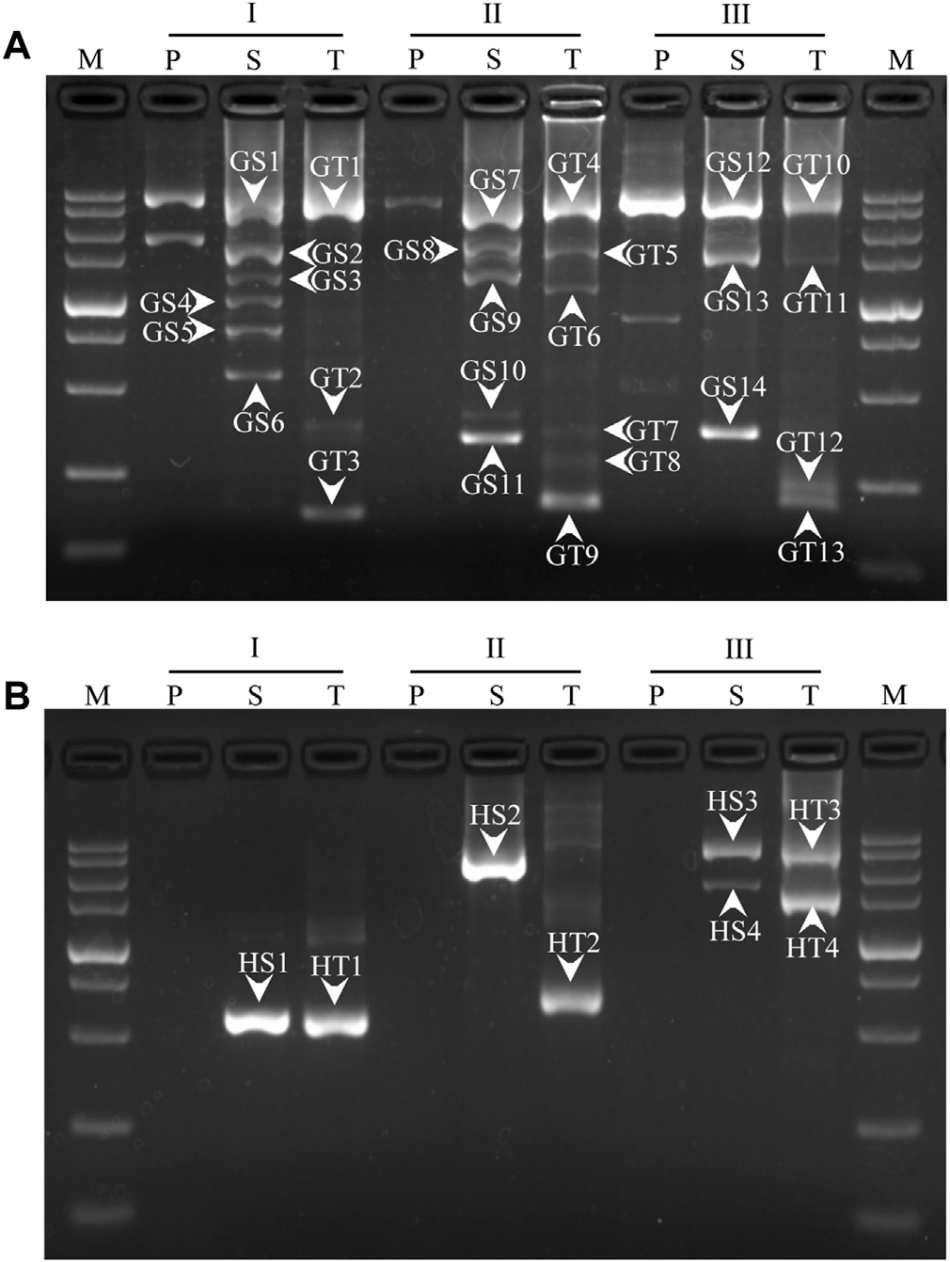
Genome walking for *gadA* of *L. brevis* CD0817 **(A)** and *hyg* of rice **(B)**. I, II, and III represent three sets of WW-PCRs in a walking, individually participated by the three WWP permutations, as indicated in Table 2; lanes P, S, and T represent primary, secondary, and tertiary PCRs, respectively; bands GS1-GS14 and GT1-GT13 indicate secondary and tertiary PCR products for *gadA*, respectively; and HS1-HS4 and HT1-HT4 indicate secondary and tertiary PCR products for *hyg*, respectively. The band numbers correspond to those in Supplementary Figure S1. M: DL 5000 DNA Marker (5000, 3000, 2000, 1500, 1000, 750, 500, 250, and 100 bp).

The PCR products were separated by agarose electrophoresis. As shown in Figure 2, each WW-PCR released discrete DNA band(s) after two or three rounds of reactions. The distinct bands in secondary and tertiary PCRs were recovered for T-cloning and sequencing. Sequence alignment was conducted using the MegAlign tool in Lasergene software. The results demonstrated that all of the bands belong to target products as they are identical to the corresponding reference sequence (Supplementary Figure S1). In most cases, more than one clear DNA band appeared in a PCR (secondary/tertiary). Figure 2 also demonstrates that each WW-PCR in the same set exhibited a distinctive electrophoretic pattern, and the longest amplicon ranged from 1 to 3 kb in size. Specifically, the largest fragments walked for *gadA* by the three WW-PCRs were 2.8 (GT1), 3.0 (GT4), and 3.3 kb (GT10), respectively (Figure 2A), and for *hgy* were 0.6 (HT1), 0.7 (HT2), and 3.1 kb (HT3), respectively (Figure 2B).

## Discussion

In this study, the WW-PCR, a new tool for determining unknown flanking DNA, has been established. The key to WW-PCR is the use of WWPs characterized by identical 5′-part and 3′-part and heterologous spacers. The current technique was termed WW-PCR due to the formation of wristwatch-like structures between WWPs. We have illustrated how the WW-PCR can be used to efficiently obtain unknown flanking regions, starting from a known DNA sequence (Figure 1). A WWP used in primary PCR determines the annealing pattern, while the one used in the secondary or tertiary PCR is responsible for eliminating non-target products.

The 3′-overlap ensures that the WWP initiates DNA extension once it anneals to the former WWP complement, with the 5′-overlap stabilizing the wristwatch-like structure (Tan et al., 2005). In general, functional priming requires at least a 2-nt accurate match at the 3′-end (Parker et al., 1991; Parks et al., 1991). A longer identical 3′ end (five or more bases), however, may weaken individualized random annealing of the WWPs in the primary PCR (Parks et al., 1991). Comprehensively, a 3′-overlap of 3 nt was assigned to the WWPs in this study. The overall difference in the sequence, due to the 10 nt mismatch, facilitates personalized annealing of the WWPs in the primary PCR, providing a guarantee for the success and efficiency of the WW-PCR. It can be expected that if more than one WWP permutation is used, at least one would give positive results, and some may produce satisfactory amplicon(s). In this work, each WW-PCR yielded positive outcomes (Figure 2), suggesting a high success rate of the WW-PCR. The longest product from each walking was approximate 4 kb (Figure 2), verifying the high efficiency of the WW-PCR. In most cases, multiple bands were observed in a secondary/tertiary PCR (Figure 2). This is common in PCR-based genome walking as the primary walking primer has multiple annealing sites on the flank of interest (Chang et al., 2018; Lo et al., 2018). We also noticed that a DNA band of the secondary PCR was slightly larger than that of the corresponding tertiary PCR (Figure 2), which is attributed to the mutual position relationship between the nested GSPs used (Liu et al., 2007).

In the RSC of the secondary/tertiary PCR, in the case that the WWP is well-matched with some site(s) internal to the former WWP locus on an unknown region, the WWP may anneal to the site and prime DNA elongation. If this annealing occurs on the DNA of interest, an extra shorter target product may be generated. If this annealing occurs on the non-target DNA, the resultant shorter one cannot be further amplified in the subsequent HSCs because it lacks a perfect binding site for any primer (Figure 1). Therefore, the internal annealing of the WWP contributes to the multi-band phenomenon while not affecting the specificity of the PCR. It should be pointed out that the DNA band pattern of any tertiary PCR resembles with that of the corresponding secondary PCR (Figure 2), implying that internally partial annealing is rare.

It is worth emphasizing that the Tm between WWPs should be at least 20°C lower than that of any WWP itself (Li et al., 2015; Chang et al., 2018) so that the WWP anneals to the former WWP locus only in the one RSC of the secondary/tertiary PCR (Figure 1). Here, the Tm between the WWPs was as low as around 40°C, while that of any WWP itself is rather high (60–65°C) (Table 1). The sequences of WWPs are variable, as long as they can form a wristwatch-like structure under expected temperature. Moreover, users can devise x WWPs as their wish to perform x sets of WW-PCRs. The three WWPs (Table 1) presented here have been validated. Users need to design their nested GSPs according to known sequences. The sequences of our WWPs are completely random and thus should be universal for any genomes.

Non-target amplification is a big problem in PCR-based walking strategies (Kilstrup et al., 2000). Three types of non-target products are usually produced: I) primed by GSP alone, II) primed by GSP and random primer (here referred to as WWP), and III) primed by the random primer alone (Arnold et al., 1991; Bae et al., 2010; Wang et al., 2011), as shown in Figure 1. Types I and II could be easily diluted in the next round of the PCR as there is a lack of an authentic binding site for the inner GSP. The real challenge presented is the elimination of type III products (Thirulogachandar et al., 2011; Zhu et al., 2016). The WW-PCR can effectively inhibit the amplification of type III. In each WW-PCR, the partial annealing of the WWP in the one LSC/RSC directs the synthesis of a new non-target ssDNA. This nascent ssDNA and its template, however, cannot be further amplified in the following HSCs due to their lack of complementary sites for any primers. Our results confirmed the high specificity of WW-PCR, as all the clear bands in the secondary or tertiary PCR were correct (Figure 2).

For the inverse PCR or cleavage-ligation-mediated PCR, extra operations are compulsory prior to the amplification reaction, sequentially including restriction digestion, self-cyclization, or ligation of the adapter/linker/cassette to target DNA. These steps are time-consuming, expensive, and are always accompanied by a strong background (Leoni et al., 2008; Reddy et al., 2008; Deng et al., 2010). The randomly primed strategy does avert these extra steps prior to the PCR. The reproducibility, efficiency, or universality of the available randomly primed PCRs, however, has been unsatisfactory (Wang et al., 2007; Zhou et al., 2012; Wang et al., 2013). Compared to the routine randomly primed methods, WW-PCR may have at least one of the following advantages: 1) great simplicity and efficiency with more than one set of the WW-PCR that can be set up by simply varying the use order of the WWPs, increasing the success rate and efficiency of DNA walking; 2) superior versatility with the WWPs being universal for any genomes as they are completely random; and 3) high specificity with the WW-PCR selectively accumulating target DNA while removing non-target species as any primers partially anneal to the DNA template once only. A detailed comparison of the WW-PCR to the existing classical walking methods is shown in Table 4.

**TABLE 4:** Comparison of different PCR-based genome walking methods.

Method	Principle	ESMFS	ESMFE	References
Inverse PCR	DNA is digested and then self-cyclized. The cyclized DNA is subjected to the PCR driven by two GSPs facing in opposite directions.	No	No	Ochman et al. (1988), Kotik (2009)
CL-PCR	DNA is digested and then ligated to a synthetic oligo. The ligated DNA undergoes two-three rounds of nested PCRs performed by the oligo primer sequentially pairing with nested GSPs.	No	No	Mueller et al. (1989), Kotik (2009)
TAIL-PCR	A short degenerate primer with low Tm is used as the walking primer. The one LSC is included in every three cycles to facilitate walking primer annealing. A target product is preferentially enriched due to its higher amplification efficiency than a non-target one.	Yes	Yes	Liu et al. (1995), Kotik (2009)
POP-PCR	A set of POPs with 3′-overlap are paired with nested GSPs. POP partially anneal to the DNA template in the one LSC/RSC of each PCR, producing a pool of ssDNAs. The target ssDNA is converted into dsDNA by the GSP in the next HSC, while the non-target one cannot be converted into dsDNA.	Yes	Yes	Li et al. (2015)
WW-PCR	The principle is presented in the section “Overview of WW-PCR” of this study.	Yes	Yes	This study

Note: GSP, gene-specific primer; CL-PCR, cleavage-ligation-mediated PCR; TAIL-PCR, thermal asymmetric interlaced PCR; POP-PCR, partially overlapping primer-based PCR; WW-PCR, wristwatch PCR; ESMFS, extra safeguard mechanism for success; ESMFE, extra safeguard mechanism for efficiency; LSC, low-stringency cycle; RSC, reduced-stringency cycle; HSC, high-stringency cycle.

The targeted long-read sequencing method has gained substantial interest as an emerging technology (Bethune et al., 2019; Xin et al., 2021). However, the high error rate and high cost increase the difficulty of promoting this next-generation technology at this stage (Mitsuhashi et al., 2020; Ebert et al., 2021). The WW-PCR is currently more adaptable for a general laboratory, given its cheapness and high accuracy. Meanwhile, in the future, the WW-PCR may become a supplement to the targeted long-read sequencing.

## Conclusion

The WW-PCR, an efficient and reliable genome walking tool based on the partial overlap between WWPs, has been described in this work. The concept of the WW-PCR has been validated in the genomes of a microbe and rice. The current method is a promising alternative to the existing genome walking technologies because of its specificity, simplicity, and efficiency.

## Data Availability

The datasets presented in this study can be found in online repositories. The names of the repository/repositories and accession number(s) can be found at: https://www.ncbi.nlm.nih.gov/genbank/, AYM03982.1 https://www.ncbi.nlm.nih.gov/genbank/, and KF206149.
