# Supplementary material for: Wristwatch PCR: A Versatile and Efficient Genome Walking Strategy
Source: Front Bioeng Biotechnol. 2022 Apr 12;10:792848. doi: 10.3389/fbioe.2022.792848 (PMC9039356; doi:10.3389/fbioe.2022.792848)
Supplement: Supplementary file 1 [file Table1.DOCX]

**a**

**Unknown Region**

**Known Region**

***gadA*SP2**


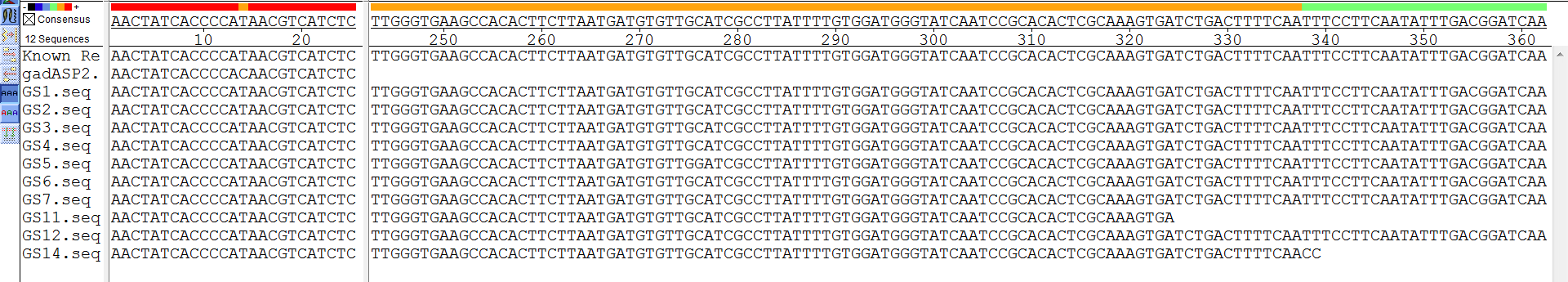
**b**

**Unknown Region**

***gadA*SP3**

**Known Region**


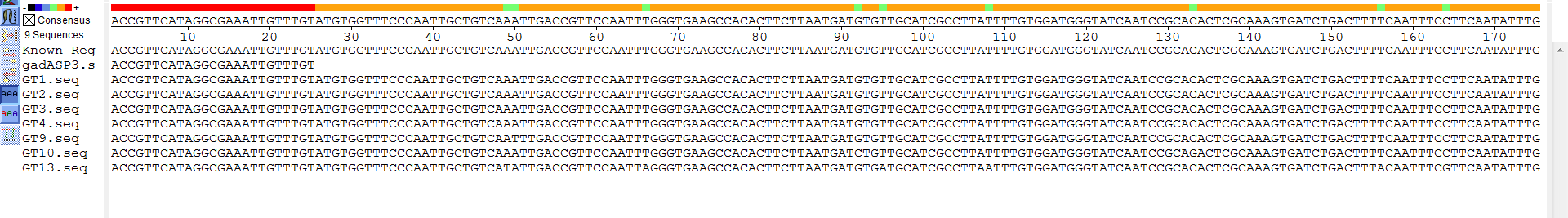


**c**

**Unknown Region**

**Known Region**

***hyg*SP2**


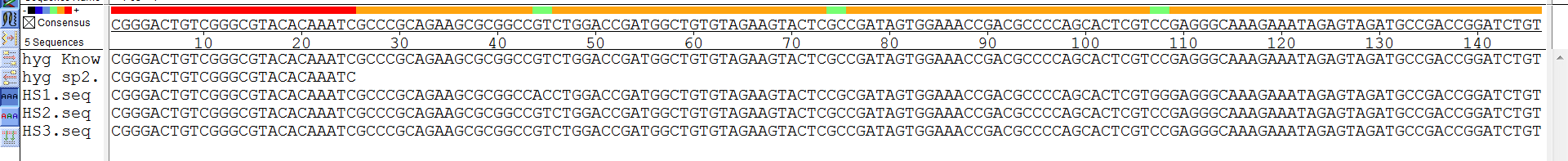


**d**

**Unknown Region**

**Known Region**

***hyg*SP3**


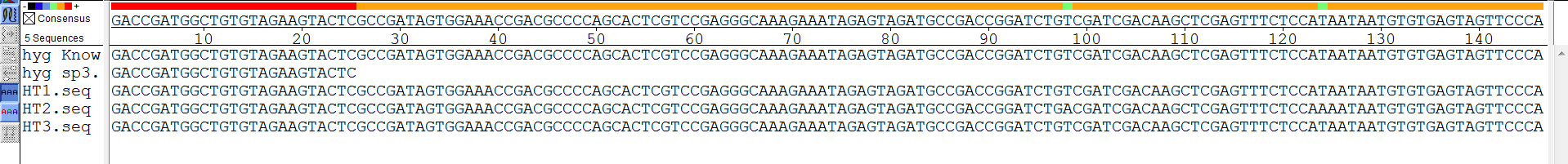


**Figure S1 | Sequence alignment** **of the *gadA* locus in *L. brevis* CD0817 and *hyg* in rice.** a: the sequence alignment of the secondary PCR clear bands in *gadA*; b: the sequence alignment of the tertiary PCR clear bands in *gadA*; c: the sequence alignment of the secondary PCR clear bands in *hyg*; d: the sequence alignment of the tertiary PCR clear bands in *hyg*; red box indicates the amplified region.
